# Focus on necroptosis: its role in the pathogenesis and therapeutic potential of osteoarthritis

**DOI:** 10.3389/fimmu.2026.1695254

**Published:** 2026-03-30

**Authors:** Yuhong Ouyang, Haiyang Liao, Wenjiao Kang, Fengyuan Li, Haili Shen

**Affiliations:** 1 The Second Clinical Medical College of Lanzhou University, Lanzhou, China; 2 Department of Rheumatology, Lanzhou University Second Hospital, Lanzhou, China

**Keywords:** MLKL, necroptosis, osteoarthritis (OA), programmed cell death (PCD), RIPK1

## Abstract

Osteoarthritis (OA) is a prevalent degenerative joint disorder characterized by the progressive deterioration of joint structures. This deterioration results in pain and functional impairment and is a significant contributor to disability and economic strain. Necroptosis represents a crucial form of programmed cell death (PCD) that operates independently of caspases; it is governed by receptor-interacting protein kinase 1 (RIPK1), RIPK3, and mixed lineage kinase domain-like protein (MLKL). Necroptosis plays a pivotal role in various inflammatory, infectious, and degenerative pathologies. Recent research studies have highlighted the significant involvement of necroptosis in the advancement of OA. This article delineates the processes underlying the initiation and execution of necroptosis and explores its potential mechanisms within OA. It emphasizes the interplay between necroptosis and oxidative stress, inflammatory responses, extracellular matrix degradation, and cartilage repair and regeneration. Ultimately, this review assesses the existing evidence regarding the potential of targeting necroptosis as a therapeutic avenue for OA, positing that inhibition of the necroptosis pathway may emerge as a novel strategy to mitigate symptoms of OA and impede the progression of joint degeneration.

## 1 Introduction

Osteoarthritis (OA) is an ordinary chronic degenerative joint ailment, marked by the degradation of articular cartilage, formation of osteophytes, and inflammation of the synovium (1). OA predominantly affects weight-bearing joints and commonly utilized joints, including the knee, hip, spine, and finger joints, with severe cases potentially leading to joint malformation and disabilities (2, 3). It is estimated that around 500 million individuals globally are afflicted by OA (4). With the aging demographic, the prevalence of this condition is increasing, imposing a significant economic strain on both patients and society (5, 6). Presently, clinical management of OA primarily encompasses pharmacological interventions, physical therapy, and surgical procedures. Although these modalities provide some symptomatic relief, they fail to fundamentally halt disease progression and are accompanied by specific side effects and limitations (7, 8). Consequently, extensive investigation into the pathophysiology of OA and the identification of novel therapeutic targets and strategies are of considerable clinical and societal importance.

Traditionally, the etiology of OA has been primarily associated with factors such as aging, mechanical wear and tear, obesity, and trauma, which lead to cartilage degeneration and repair complications (9, 10). However, emerging research increasingly underscores the pivotal role of inflammation in the evolution of OA, with necroptosis, as a novel programmed cell death mechanism, exhibiting distinct molecular regulatory pathways and inflammatory features, thus becoming a focal point in OA pathogenesis studies (11). Necroptosis was first identified as a cell death pathway activated under conditions where apoptosis is inhibited; it exhibits necrotic features, such as rupture of the cell membrane, cellular swelling, and release of intracellular contents, while simultaneously possessing programmed death characteristics that are precisely regulated by specific signaling pathways (12). Investigations reveal that dysregulation of necroptosis can influence various physiological and pathological processes, including embryonic development, immune responses, neurodegenerative diseases, and tumorigenesis (13–15). Additionally, growing evidence indicates that necroptosis plays a significant role in OA. In OA, aberrant activation of necroptosis may result in increased chondrocyte apoptosis, expedited ECM degradation, and enhanced secretion of inflammatory mediators, thus facilitating the progression of OA (16, 17). Therefore, we summarize the molecular mechanisms of necroptosis and its role in OA, and explore new avenues for the prevention and treatment of OA in conjunction with the mechanisms of necroptosis inhibitors.

## 2 Overview of necroptosis

Necroptosis was initially characterized by Degterev et al. at Harvard University in 2005 as a regulated form of cell death exhibiting necrotic morphological traits (18). Distinct from apoptosis and other programmed cell death modalities, necroptosis is characterized as a passive, non-programmed cell death typically instigated by severe cellular injury or irreversible stimuli, such as physical or chemical factors. This process results in the rupture of the cell membrane and the swift release of cellular components, which incites a robust inflammatory response (19, 20). Necroptosis integrates features from both apoptosis and necrosis; it is a regulated cell death modality activated by extracellular signals (for instance, the binding of death receptors to their ligands) or intracellular stimuli (such as the presence of foreign microbial nucleic acids) when apoptosis is impeded (21). The morphological characteristics of necroptosis include cell swelling, loss of membrane integrity, and eventual rupture, culminating in the release of cellular contents that incite local inflammatory reactions, mirroring the morphological aspects of necrosis. Mechanistically, necroptosis is stringently regulated by a cascade of intracellular signaling molecules, akin to the gene-regulated nature of apoptosis (22, 23). The activation of necroptosis is independent of caspase activity and necessitates the phosphorylation of mixed lineage kinase domain-like protein (MLKL), which is contingent upon receptor-interacting protein kinase 3 (RIP3). This phosphorylation triggers the formation of pore complexes on the plasma membrane, resulting in cellular lysis and the initiation of necroptosis (24).

### 2.1 RIPs as important regulatory molecules of necroptosis

The receptor-interacting protein (RIP) family comprises a group of serine/threonine protein kinases that are crucial for the necroptosis process, with RIPK1 and RIPK3 being the most significant members. RIPK1, a pivotal member of the RIP family, initiates the necroptosis signaling cascade (25, 26). It encompasses multiple domains, including an N-terminal kinase domain, a central death domain (DD), and various protein interaction domains at the C-terminus (27). The kinase activity of RIPK1 is essential for the commencement of necroptosis; upon exposure to death signals, its death domain can interact with the death domains of receptors such as tumor necrosis factor receptor 1 (TNFR1), facilitating its recruitment to the death receptor complex (28). For instance, when cells are stimulated by TNF-α, TNFR1 binds to TNF-α and subsequently recruits TRADD (TNFR1-associated death domain protein), which in turn brings RIPK1 into the fold to form complex I. Within this complex, RIPK1 undergoes several post-translational modifications, including ubiquitination, which significantly modulates its functions (29, 30). Typically, the ubiquitination of RIPK1 activates the NF-κB signaling pathway, which fosters cell survival and inflammatory responses; however, under particular circumstances—such as the destabilization of complex I or the inhibition of RIPK1 ubiquitination—RIPK1 undergoes deubiquitination and dissociates from complex I, subsequently recruiting RIPK3 to activate the necroptosis signaling pathway (31–33).

RIPK3, another serine/threonine protein kinase within the RIP family, is integral to the execution phase of necroptosis. It features distinctive structural attributes, with its kinase domain bearing some resemblance to that of RIPK1 while also exhibiting specific differences (34). The activation of RIPK3 is contingent upon its interaction with RIPK1; when RIPK1 recruits RIPK3, the two kinases engage through their respective RHIM (receptor-interacting protein homotypic interaction motif) domains to form the necrosome (35, 36). Within this necrosome, RIPK1 phosphorylates RIPK3, thereby activating its kinase activity. Activated RIPK3 subsequently phosphorylates downstream substrate molecules, with MLKL being the most critical of these targets (37).

Additionally, RIPK1 and RIPK3 exhibit cooperative functions within the necroptosis pathway, collectively influencing cellular outcomes. The interactions between these proteins are meticulously regulated by various elements, including caspase-8 and FLIP (FLICE-like inhibitory protein) (38, 39). Caspase-8 impedes necroptosis through the cleavage of RIPK1 and RIPK3, while FLIP collaborates with caspase-8 to modulate its activity, subsequently impacting the equilibrium between necroptosis and apoptosis (40). Moreover, certain intracellular signaling molecules and metabolic byproducts may also engage in the regulatory dynamics of RIPK1 and RIPK3, making the regulatory mechanisms of necroptosis more complex (41, 42).

### 2.2 Molecular mechanisms of the necroptosis pathway

#### 2.2.1 Key regulatory proteins

RIPK1 possesses a dual role and intricate regulatory function within the necroptosis process. At the onset of necroptosis signaling, RIPK1 serves as a pivotal initiating element for signal transduction by associating with the death receptor complex via its death domain, which triggers downstream signaling cascades. The kinase activity of RIPK1 is essential for the commencement of necroptosis; without it, the signaling pathway for necroptosis cannot be effectively initiated (43, 44). Nevertheless, RIPK1’s involvement extends beyond necroptosis; under standard physiological states, it activates the NF-κB signaling pathway through ubiquitination, thereby supporting cell survival and inflammatory responses (45, 46). It is only under specific circumstances, such as the inhibition of the apoptotic pathway, that RIPK1 transitions to activate the necroptosis signaling pathway, illustrating its complex regulatory mechanism and establishing RIPK1 as a crucial “molecular switch” in determining cellular survival or death (47, 48).

RIPK3 functions as the central protein during the execution phase of necroptosis. It engages with RIPK1 via the RHIM domain to form the necrosome, where it undergoes phosphorylation and activation by RIPK1 (49). Once activated, RIPK3 exhibits kinase activity, enabling it to specifically phosphorylate MLKL, thus facilitating the downstream transmission of the necroptosis signal (50, 51). Research has indicated that cells or animal models lacking the RIPK3 gene fail to appropriately activate the necroptosis signaling pathway even when exposed to potent necroptosis-inducing stimuli, underscoring the essential role of RIPK3 in the execution of necroptosis (52, 53).

MLKL represents the final component in the necroptosis cascade; in its inactive form, MLKL resides in the cytoplasm as a monomer (54, 55). Upon activation and phosphorylation by RIPK3, MLKL undergoes oligomerization, and structural alterations allow it to interact with membranes (56). The oligomerized MLKL migrates to the plasma membrane, where it forms pores that compromise membrane integrity, culminating in the release of cellular contents and the subsequent induction of necroptosis. Thus, the activation and translocation of MLKL to the membrane are regarded as critical events in the necroptosis process (57).

Caspase-8 acts as a significant negative regulator of necroptosis; it inhibits the necroptosis process by cleaving RIPK1 and RIPK3 (58). Within complex IIa, when caspase-8 is activated under normal conditions, it cleaves RIPK1 and RIPK3, thereby preventing necrosome formation and steering the cell towards apoptosis. Conversely, if caspase-8 activity is compromised, the necroptosis signaling pathway can be activated (59, 60).

ZBP1 also assumes a distinctive role in necroptosis. It can detect mitochondrial DNA/RNA and other cytoplasmic elements, and under specific stress conditions, such as viral infections or nutrient deprivation, ZBP1 interacts with RIPK1 and RIPK3 via its RHIM domain to directly instigate the necroptosis signaling pathway (61, 62). The action of ZBP1 is independent of death receptor signaling, introducing a novel mechanism for the initiation of necroptosis (63).

#### 2.2.2 Major signaling pathways

Among the many signaling pathways involved in necroptosis, the RIPK1-RIPK3-MLKL signaling cascade triggered by the interaction of TNF-α with TNFR1 represents one of the most fundamental and pivotal pathways in cellular signaling (64, 65). Upon binding of TNFα to TNFR1, the intracellular domain of TNFR1 undergoes a conformational alteration. This change facilitates the recruitment of TRADD, thereby forming the initial signaling complex. Following this event, TRADD interacts with RIPK1 through its Death Domain, resulting in the assembly of the TNFR1-TRADD-RIPK1 complex, commonly referred to as complex I (29). Within this complex, RIPK1 undergoes a variety of post-translational modifications, notably linear ubiquitination mediated by the linear ubiquitin chain assembly complex (LUBAC), while cIAP1/2 also associate with complex I. In this modified state, RIPK1 predominantly activates the NF-κB signaling pathway, thereby enhancing cell survival and promoting the expression of genes associated with inflammation (66, 67).

However, when the stability of complex I is compromised, such as when cIAP1/2 are degraded, RIPK1 undergoes deubiquitination and dissociates from complex I (68). At this point, RIPK1 recruits FADD and caspase-8 to form complex IIa. If caspase-8 is normally activated, the cell will proceed towards apoptosis, but in cases where the apoptotic pathway is blocked, for example, when the activity of caspase-8 is inhibited, RIPK1 recruits RIPK3 (69). RIPK1 and RIPK3 interact through their RHIM domains to form the necrosome. In the necrosome, RIPK1 exerts kinase activity, phosphorylating RIPK3, thereby leading to RIPK3 activation (70).

Activated RIPK3 phosphorylates MLKL as its important downstream substrate. After MLKL is phosphorylated by RIPK3, its conformation changes from a monomer to an oligomer (71). Oligomerized MLKL has stronger membrane affinity and translocates from the cytoplasm to the cell membrane. On the cell membrane, MLKL oligomers form pore structures, increasing the permeability of the cell membrane (72). A large influx of ions such as sodium and calcium occurs, leading to an osmotic imbalance within the cell and cell swelling. As swelling intensifies, the cell membrane ultimately ruptures, releasing cellular contents into the extracellular environment, triggering a strong inflammatory response, thus completing the process of necroptosis (73, 74).

**Figure 1:**
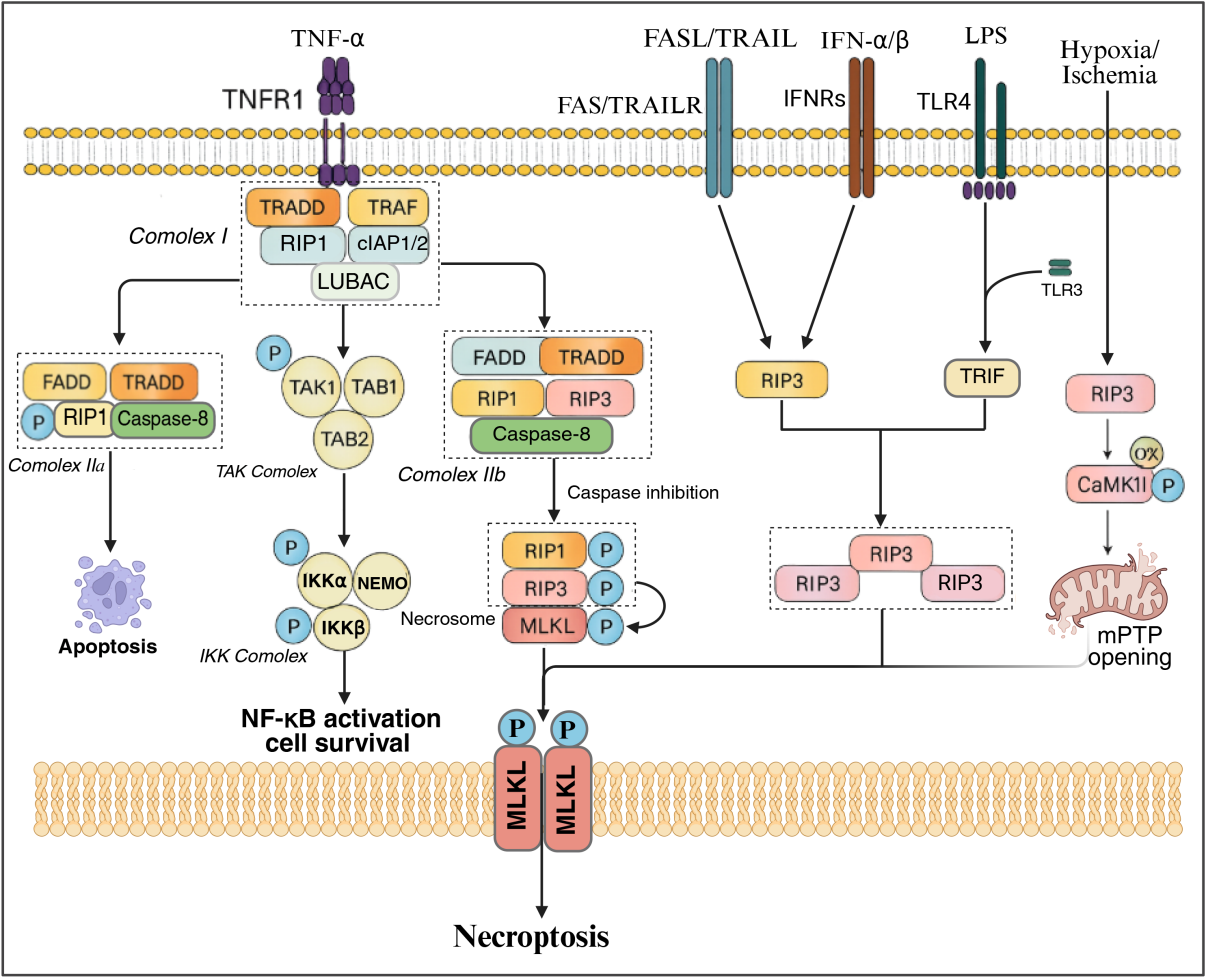
The molecular underpinnings of necroptosis involve not only the conventional TNF/TNFR1-mediated signaling cascade but also the engagement of various other receptors such as FASL, TRAIL, IFNα/β, and LPS. These ligands trigger their specific receptors, consequently activating the TRIF and RIPK1 signaling pathways. Following this activation, the receptors interact with RIPK3 via their RHIM (receptor interacting protein homotypic interaction motif) domains, which facilitates the activation of RIPK3. Once activated, RIPK3 phosphorylates MLKL, leading to its oligomerization, integration into the membrane, and disruption of intracellular membranes, ultimately culminating in the process of necroptosis. Additionally, hydrogen peroxide has been shown to stimulate the production of ROS, which subsequently induces mitochondrial dysfunction through interactions with mitochondrial proteins and activation of the PARP-AIF pathway, thereby promoting necroptosis.

Moreover, recent investigations have demonstrated that beyond the conventional TNFα-mediated necroptosis, RIPK1 and RIPK3 are also capable of activating mitochondrial stress pathways that mediate necroptosis. Examples of these pathways include the RIP1-mediated RIP1-MLKL-MEK/ERK pathway, as well as RIP3-mediated pathways such as RIP3-cJun N-terminal kinase (JNK)-BNIP3, RIP3-PGAM5-Drp-1, and RIP3-calmodulin-dependent protein kinase II (CaMKII)-mediated mitochondrial permeability transition pore (mPTP) pathways (75, 76) (**Figure 1**).

## 3 The impact of necroptosis on the pathogenesis of OA

### 3.1 Necroptosis and oxidative stress

Oxidative stress serves as a potential trigger for the activation of the necroptosis signaling pathway. Under conditions of oxidative stress, certain intracellular signaling molecules, such as mitogen-activated protein kinases (MAPK) and nuclear factor-κB (NF-κB), become activated (77, 78). These signaling molecules can further modulate the expression and functionality of proteins implicated in necroptosis (79). Research indicates that ROS may activate RIPK1, thereby initiating the necroptosis signaling pathway (80). Additionally, ROS may affect the activity of caspase-8. When caspase-8 is inhibited, RIPK1 binds to RIPK3, leading to complex formation that further activates MLKL. This culminates in pore formation in the plasma membrane and subsequent necroptosis (81).

Oxidative stress, caused by excessive ROS production, has been shown to play an important role in the progression of OA (82). Specifically, in the pathophysiology of OA, the oxidative stress response in cells can lead to necroptosis of chondrocytes, which not only exacerbates the inflammatory response in the joints but also further promotes cartilage degeneration and damage (83). Moreover, the process of necroptosis can exacerbate oxidative stress. When cells undergo necroptosis, the cell membrane ruptures, releasing cellular contents, some of which—such as damage-associated molecular patterns (DAMPs)—may further induce ROS production (84). Inflammatory factors produced during necroptosis can also stimulate cells to produce more ROS (85). For example, TNF-α can activate NADPH oxidase, promoting ROS generation. This exacerbates oxidative stress and promotes necroptosis (86, 87). This vicious cycle between oxidative stress and necroptosis leads to continuous damage to joint tissues, thereby promoting the development of OA (88, 89). High levels of ROS and the expression of necroptosis-related proteins are often detected in the articular cartilage and synovial tissues of OA patients, further confirming the close relationship between the two in the pathogenesis of OA (90, 91).

### 3.2 Necroptosis and inflammatory response

Necroptosis and the inflammatory response are intricately linked in the pathophysiology of OA. Together, they establish a multifaceted network that collaboratively enhances the progression of the disease (92). Necroptosis is characterized as a form of programmed cell death that exhibits pro-inflammatory properties. In the context of OA, when joint cells, including chondrocytes and synovial cells, undergo necroptosis, they release intracellular components into the extracellular environment (93). These components encompass DAMPs such as high mobility group box 1 (HMGB1) and mitochondrial DNA (94, 95). DAMPs are identified by pattern recognition receptors (PRRs) present on immune cells, which activate these immune cells and initiate inflammatory responses (96, 97). HMGB1, in particular, interacts with Toll-like receptors (TLRs) and thereby activates the NF-κB signaling pathway (98, 99). Consequently, this activation results in the upregulation of gene expression and secretion of inflammatory mediators such as TNF-α, IL-1β, and IL-6. These mediators further stimulate additional immune cells, including macrophages and lymphocytes, prompting them to release more inflammatory agents and thus establishing an inflammatory cascade. Moreover, this cascading process not only intensifies joint pain and dysfunction but may also expedite cartilage degradation and synovial inflammation, exacerbating the overall condition (100, 101).

Within the OA joint microenvironment, an array of inflammatory mediators, including TNF-α and Fas ligand, is present (102). These mediators have the capacity to bind to death receptors located on the cellular surface, thus initiating the necroptosis pathway (103). Upon the binding of TNF-α to tumor necrosis factor receptor 1 (TNFR1), proteins such as RIPK1 are recruited, leading to the formation of a signaling complex. In scenarios where the apoptotic pathway is inhibited, RIPK1 subsequently interacts with and phosphorylates RIPK3, which in turn activates MLKL, culminating in necroptosis (104). Additionally, inflammatory factors can enhance cellular sensitivity to necroptosis by modulating specific intracellular signaling pathways, such as the NF-κB pathway, and metabolic processes (105). For instance, IL-1β can upregulate the expression of RIPK3, thereby increasing the susceptibility of cells to necroptosis (106). The interplay between necroptosis and the inflammatory response aggravates the localized inflammatory condition within the joints, thereby promoting cartilage degeneration and joint tissue damage, which propels the progression of OA (107).

### 3.3 Necroptosis and extracellular matrix degradation

In the pathogenesis of OA, necroptosis is closely related to ECM degradation, as necroptosis leads to the destruction of the ECM through various pathways; thereby affecting the normal function of articular cartilage (108). The ECM of articular cartilage is mainly composed of type II collagen, proteoglycans, and other components, which are crucial for maintaining the structural integrity and mechanical properties of cartilage (109). When chondrocytes undergo necroptosis, some enzymes and signaling molecules are released into the extracellular space. These substances, by interacting with cellular receptors and signaling cascades, activate the expression and activity of a series of matrix-degrading enzymes, among which the matrix metalloproteinase (MMP) family plays a key role in ECM degradation (110).

Research indicates that inflammatory signals related to necroptosis trigger the NF-κB signaling pathway within chondrocytes (111). When NF-κB translocates into the nucleus, it associates with the promoter regions of MMP genes, thereby enhancing their transcription and subsequent expression (112). Notably, MMP-13 has the capability to specifically degrade type II collagen, resulting in diminished mechanical properties of cartilage and jeopardizing its structural integrity (113). Moreover, proteoglycanases, particularly those belonging to the ADAMTS family, exhibit increased expression levels when stimulated by necroptosis (114). ADAMTS-4 and ADAMTS-5 are responsible for the degradation of proteoglycans, leading to a loss of elasticity and water-holding capacity in cartilage, which further accelerates its degeneration (115). Additionally, necroptosis may impair the synthesis of ECM components by influencing the metabolic functions and biosynthetic capacity of chondrocytes (116). Following necroptosis, the metabolic activities of chondrocytes are suppressed, which leads to a decline in their capacity to synthesize ECM components, including type II collagen and proteoglycans. This disruption in the balance between synthesis and degradation of the ECM exacerbates the degradation process and contributes to cartilage degeneration (117). In the articular cartilage of OA patients, significant ECM degradation occurs around necroptotic chondrocytes, underscoring the pivotal role of necroptosis in ECM degradation and the overall pathogenesis of OA (118).

### 3.4 Necroptosis and cartilage regeneration and repair

**Figure 2:**
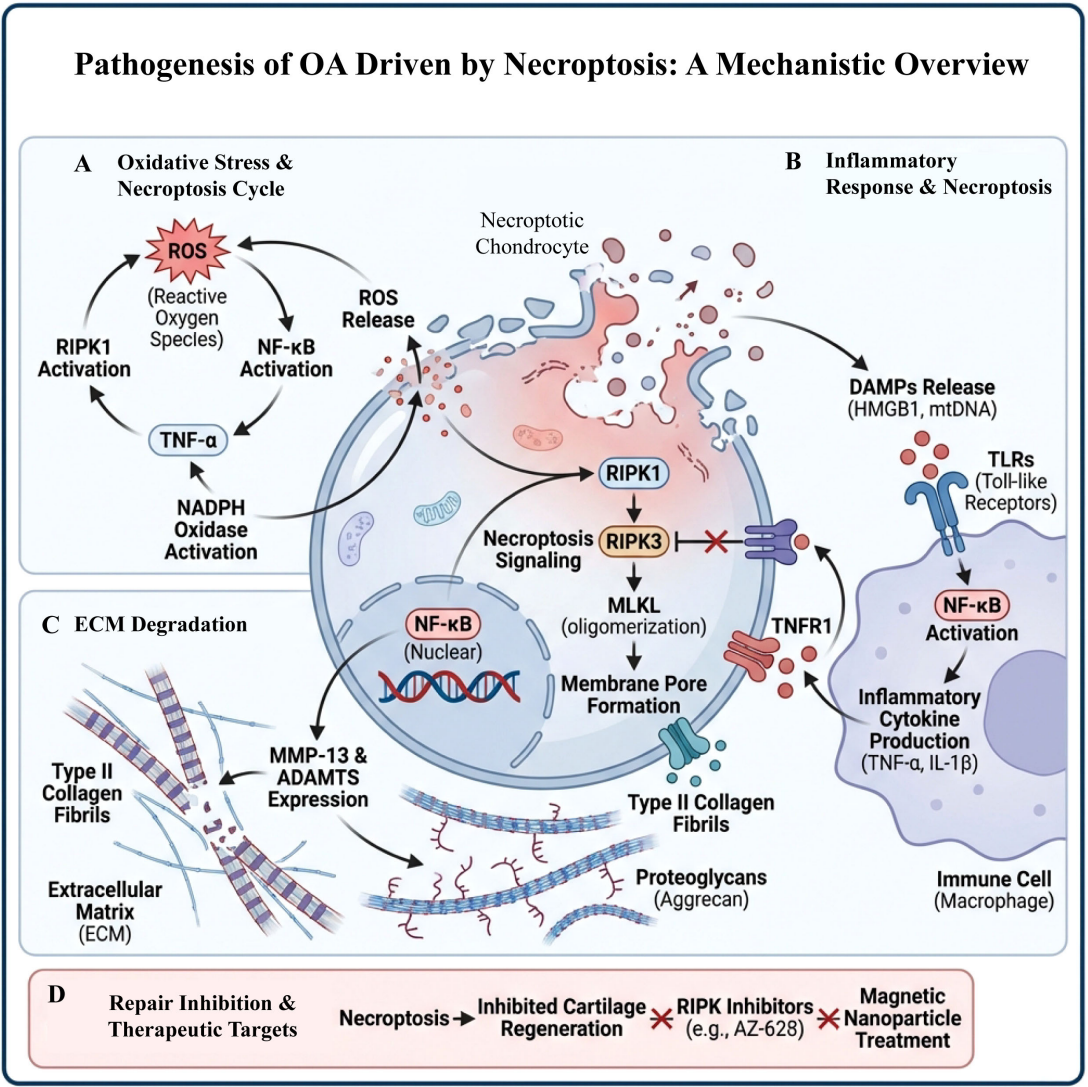
Schematic diagram of the pathogenesis of necroptosis-driven osteoarthritis. This figure outlines the role of necroptosis in OA from four aspects: oxidative stress, inflammatory response, matrix degradation, and therapeutic targets. **(A)** Oxidative stress and the necroptosis cycle. After RIPK1 activation, ROS is produced via TNF-α signaling and NADPH oxidase activity. ROS further activates NF-κB and promotes the release of inflammatory factors, forming a positive feedback loop of “RIPK1–ROS–NF-κB–TNF-α,” thereby exacerbating chondrocyte necroptosis. **(B)** Inflammatory response and necroptosis. Necroptotic chondrocytes release DAMPs such as HMGB1 and mitochondrial DNA, activating TLRs and TNFR1 on immune cells. This activation triggers NF-κB-mediated production of inflammatory factors such as TNF-α and IL-1β, further promoting RIPK1/RIPK3/MLKL signaling activation. **(C)** Degradation of the ECM. NF-κB activation upregulates the expression of matrix-degrading enzymes such as MMP-13 and ADAMTS, leading to the continuous breakdown of type II collagen fibers and proteoglycans such as aggrecan, resulting in the destruction of the cartilage ECM. **(D)** Impaired repair mechanisms and therapeutic targets. Continuous necroptosis not only inhibits cartilage regeneration but also provides potential targets for novel interventions. These include RIPK1 pathway inhibitors (e.g., AZ-628) and drug delivery via magnetic nanoparticles. Both approaches may block the vicious cycle of necroptosis–inflammation–matrix degradation.

Necroptosis exerts a profound influence on cartilage regeneration and repair in the context of OA. These processes are significantly interconnected and jointly influence the pathological alterations of articular cartilage and the progression of the disease (119). Recent researches have demonstrated that necroptosis modulates cartilage regeneration and repair through the regulation of chondrocyte viability and the inflammatory response (120). The hallmark of necroptosis is the activation of receptor-interacting protein kinases 1 and 3 (RIPK1 and RIPK3), which culminates in the rupture of the cell membrane and the subsequent release of DAMPs. These DAMPs are instrumental in driving inflammatory processes (121). Within the pathological framework of OA, chondrocyte necroptosis is intimately linked to localized inflammatory responses (122). Evidence indicates that TNF-α can instigate the activation of RIPK1 and RIPK3, thereby facilitating chondrocyte necroptosis (123). This mechanism not only results in the demise of chondrocytes but also intensifies the inflammatory response within the joint environment, establishing a detrimental cycle that further accelerates cartilage degeneration (124). By targeting the activity of RIPK1 and RIPK3, it is feasible to reduce chondrocyte death and decrease levels of inflammatory mediators, consequently mitigating cartilage injury (103). In terms of cartilage regeneration and repair, necroptosis pathways present novel therapeutic avenues. For instance, certain RIPK3 inhibitors have demonstrated efficacy in slowing cartilage degradation in murine models of OA while concurrently decreasing the release of inflammatory factors. Furthermore, contemporary research indicates that integrative treatment approaches utilizing magnetic nanoparticles in conjunction with pulsed electromagnetic fields can attenuate the progression of OA by inhibiting necroptosis in chondrocytes (125) (**Figure 2**).

## 4 Cell types associated with necroptosis in osteoarthritis

### 4.1 Chondrocytes

Chondrocytes play a pivotal role in the development of OA, particularly in initiating necroptosis (126). Necroptosis is a specific type of programmed cell death primarily mediated by receptor-interacting protein kinases (RIPK1 and RIPK3) and mixed lineage kinase domain-like protein (MLKL). This pathway is markedly elevated in OA chondrocytes (127). In OA pathology, key necroptosis markers such as RIPK3 and phosphorylated MLKL are expressed at elevated levels in chondrocytes. This leads to disruption of cell membranes and the release of immunogenic substances, which exacerbate cartilage damage and inflammatory processes (128, 129). Furthermore, therapeutic strategies using anti-necroptosis agents have been shown to reduce RIPK3 and MLKL levels in chondrocytes, thereby slowing OA progression (130).

The alterations in necroptosis expression within OA-affected cartilage are not solely linked to heightened cell death; they also influence the remodeling of the cartilage matrix and the maintenance of the extracellular matrix’s structural integrity (131). Notably, the activation of RIPK1 is intricately associated with the degradation of the cartilage matrix and the inflammatory responses characteristic of OA. Inhibiting RIPK1 has been shown to enhance chondrocyte viability and matrix biosynthesis (132). Consequently, therapeutic approaches that target necroptosis could unveil novel avenues for OA treatment.

### 4.2 Synovial cells

Synovial cells are integral to the pathophysiology of OA, particularly in mediating synovitis and joint degradation (133). These cells predominantly consist of synovial fibroblasts and macrophages, which are responsible for synthesizing a variety of cytokines and inflammatory mediators during joint inflammation. This activity culminates in sustained synovial inflammation and exacerbates joint deterioration (134, 135). Research indicates that the necroptosis of synovial cells not only results in cellular demise but also triggers the release of DAMPs, which can subsequently activate neighboring immune cells, thereby intensifying inflammatory reactions and joint injury (136).

Receptor-interacting protein kinase 1 (RIPK1) serves as a pivotal regulator of necroptosis and significantly influences the inflammatory signaling pathways within synovial cells (137). Investigations have demonstrated that RIPK1 activation can enhance the inflammatory response in these cells by modulating downstream signaling cascades, including the NF-κB and MAPK pathways. During the pathological progression of OA, there is a marked elevation in RIPK1 expression levels in synovial cells, which correlates closely with the severity of synovial inflammation. Moreover, the inhibition of RIPK1 represents a promising therapeutic strategy for OA, as it may mitigate necroptosis of synovial cells and subsequently diminish inflammation levels, thereby decelerating joint destruction (138, 139).

The necroptosis of synovial cells not only alters the localized joint environment but may also affect the inflammatory conditions of distal tissues through the secretion of cytokines and other bioactive molecules. This inter-tissue inflammatory response could be a critical mechanism underlying the progression of OA (140, 141). Consequently, targeting the necroptosis pathway in synovial cells could not only reduce local inflammation but also elicit systemic impacts on inflammatory responses, offering novel insights and potential therapeutic avenues for OA management.

### 4.3 Osteoblasts and osteoclasts

Bone remodeling represents a dynamic physiological process that is predominantly governed by the activities of osteoblasts and osteoclasts (142). Within this context, the phenomenon of necroptosis in bone cells emerges as a significant contributor that impacts the equilibrium of bone remodeling. Research indicates that necroptosis not only compromises the viability of bone cells but may also modulate osteoclast activity via the release of pro-inflammatory mediators and cytokines, thereby resulting in an imbalance in the remodeling process (143). For instance, cell death induced by ATP has been linked to the pathogenesis of osteoporosis, highlighting that the mechanism of cell death directly influences the dynamics of bone remodeling (144). Moreover, upon undergoing necroptosis, bone cells release an array of pro-inflammatory factors, including TNF-α and RANKL, which have the potential to stimulate both the formation and functional activity of osteoclasts (145). Consequently, necroptosis in bone cells serves not merely as a mode of cell death but also as a critical regulatory mechanism in maintaining the delicate balance between bone resorption and osteogenesis.

In pathological conditions such as OA, the occurrence of necroptosis in bone cells is notably pronounced, contributing to bone loss and diminished bone density (146). Investigations have revealed that modulating the signaling pathways associated with necroptosis in bone cells—particularly the RIPK1/RIPK3/MLKL pathway—may present novel therapeutic avenues aimed at ameliorating bone health by inhibiting osteoclast formation and activity (147). Consequently, elucidating the interplay between necroptosis in bone cells and the resulting imbalance in bone remodeling holds substantial importance for the development of targeted interventions for conditions such as osteoporosis and OA.

**Figure 3:**
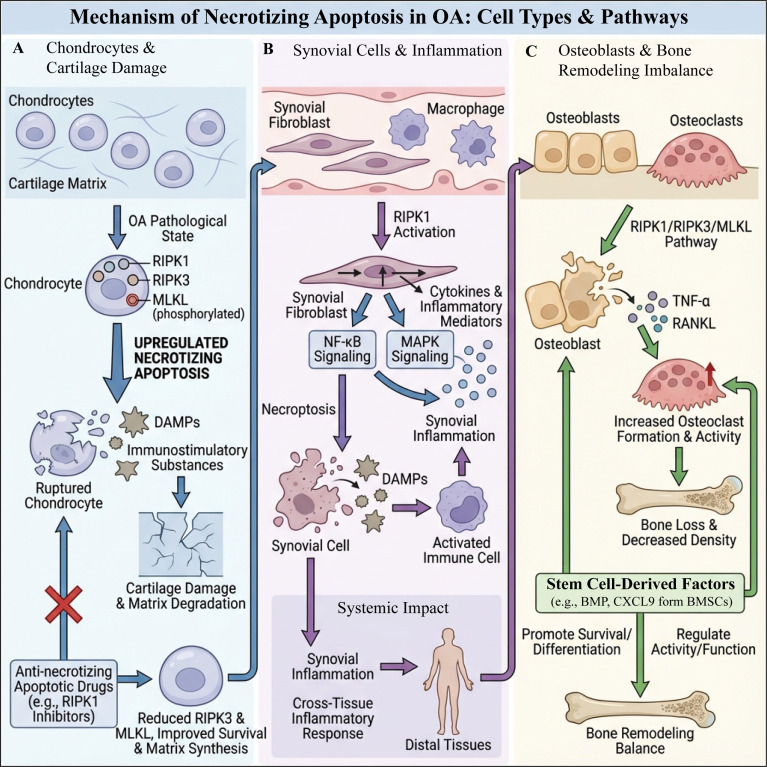
Schematic diagram of necroptosis-related cell types and pathways in OA. **(A)** (Chondrocytes and cartilage damage): In the pathological state of OA, the RIPK1–RIPK3–MLKL axis in chondrocytes is abnormally activated, leading to increased necroptosis. This results in cell rupture and the release of DAMPs and pro-inflammatory mediators, which trigger cartilage matrix degradation and structural damage. Application of necroptosis inhibitors (such as RIPK1 inhibitors) can inhibit RIPK1 signaling, thereby reducing RIPK3 and MLKL activity, enhancing chondrocyte survival and matrix synthesis. **(B)** (Synovial cells and inflammation): Activation of RIPK1 in synovial fibroblasts and macrophages drives NF-κB and MAPK signaling, producing numerous cytokines and inflammatory mediators. This induces necroptosis in synovial cells, thereby contributing to synovitis. DAMPs released from necrotic cells further activate immune cells, including macrophages and dendritic cells, amplifying local and systemic inflammation. **(C)** (Osteoblasts/osteoclasts and imbalance in bone remodeling): The RIPK1–RIPK3–MLKL pathway and TNF-α and RANKL-mediated signaling pathways promote osteoclast formation and increased activity. These effects inhibit osteogenesis as a downstream consequence, leading to decreased bone mass and an imbalance in bone remodeling. Bone marrow mesenchymal stem cell-derived factors, such as BMP and CXCL9, can promote osteoblast survival and differentiation, and regulate osteoclast activity, potentially partially restoring the balance of bone remodeling.

Moreover, factors derived from stem cells play a pivotal role in the regulation of necroptosis in bone cells. These factors exert influence over the survival and functional capacity of bone cells through a variety of mechanisms, thereby modulating the balance of bone remodeling (148). For instance, studies have demonstrated that factors secreted by bone marrow mesenchymal stem cells (BMSCs) can enhance the survival of osteoblasts while concurrently suppressing osteoclastogenesis (149). Specifically, bone morphogenetic proteins released by stem cells are recognized for their critical involvement in promoting osteoblast differentiation and function, while also exhibiting effects on osteoclast activity and functionality (150). Additionally, certain cytokines, such as CXCL9, have been observed to possess dual roles in bone metabolism, facilitating osteoblast differentiation while concurrently impacting osteoclast activity, thus serving a regulatory function in the bone remodeling process (151, 152). Thus, stem cell-derived factors not only contribute to the maintenance of bone cell survival and functionality but also exert influence over bone remodeling through the regulation of necroptosis (153) (**Figure 3**).

## 5 The impact of related signaling pathways and gene regulation on necroptosis

In the pathogenesis of necroptosis and osteoarthritis, the mitogen-activated protein kinase (MAPK) signaling pathway plays a crucial regulatory role (154). The MAPK signaling pathway mainly includes subfamilies such as extracellular signal-regulated kinase (ERK), c-Jun N-terminal kinase (JNK), and p38 MAPK (155). Notably, MAPK-related signals undergo a dynamic transition from “stress/inflammation initiation” to “sustained amplification and catabolism-death susceptibility” at different stages of OA. In the early stage of OA, joint micro-injuries lead to increased levels of inflammatory factors such as TNF-α and IL-1β. These injuries also activate mechanosensitive pathways on the cell surface and pattern recognition receptors like Toll-like receptors (TLRs) through abnormal mechanical loading/shear stress and the release of DAMPs such as cartilage matrix fragments, HMGB1, and S100 proteins. Together with inflammatory factor signals, they drive the rapid activation of MAPK, primarily reflecting the initiation of inflammatory responses and cellular stress adaptation (156). As OA progresses to the mid-stage, inflammation and oxidative stress persist, with stable activation of JNK/p38 becoming more prominent. This continuously drives inflammatory transcription and matrix degradation-related processes while lowering the cell death threshold, making chondrocytes/synovial cells more susceptible to entering the necroptosis pathway (157, 158). In the late stage of OA, under long-term high-intensity stimulation, MAPK signaling often synergistically amplifies with inflammatory hubs such as nuclear factor kappa B (NF-κB), promoting sustained high expression of inflammatory mediators and catabolic factors. This facilitates the expansion of necroptosis-related damage from focal areas to broader regions, accelerating structural destruction (159, 160). Mechanistically, when TNF-α binds to receptors on the surface of synovial cells or chondrocytes, it activates RAS protein through a series of signal transduction steps, subsequently activating RAF. RAF phosphorylates and activates MEK, which in turn phosphorylates and activates ERK (161, 162). Activated ERK can enter the nucleus to regulate the expression of related genes, promoting the production of inflammatory factors and processes such as cell proliferation and differentiation (163). Similarly, the JNK and p38 MAPK signaling pathways can also be activated by inflammatory factors, playing important roles in regulating apoptosis, inflammatory responses, and cellular stress (164). Studies have shown that inhibiting the MAPK signaling pathway can alleviate joint inflammation and cartilage damage in animal models of osteoarthritis, suggesting that MAPK plays a significant regulatory role in the pathogenesis of OA mediated by necroptosis (165).

The NF-κB signaling pathway is also indispensable in linking necroptosis and OA. NF-κB is a transcription factor that plays a key role in cellular inflammatory responses, immune regulation, and cell survival (166). Under normal conditions, NF-κB binds to the inhibitory protein IκB and exists in an inactive form in the cytoplasm. When cells are stimulated by inflammatory factors, oxidative stress, or other triggers, IκB kinase (IKK) is activated, phosphorylating IκB and promoting its degradation. The released NF-κB enters the nucleus and binds to the promoters of target genes, promoting the expression of inflammatory factors, chemokines, and cell adhesive molecules (167). From a dynamic perspective of the disease course, NF-κB primarily establishes inflammatory networks and recruits immune cells in the early stage of OA. In the mid-stage of OA, sustained inflammatory stimulation keeps NF-κB in a highly active state, not only exacerbating the inflammatory phenotype of synovial cells/chondrocytes but also potentially lowering the necroptosis threshold by affecting the balance between cell death and survival. In the late stage of OA, the release of cellular contents and damage-associated molecular patterns (DAMPs) caused by necroptosis can further activate inflammatory pathways (including NF-κB) in a feedback loop, forming a positive feedback cycle of “necroptosis—inflammatory amplification—tissue damage,” thereby promoting continuous disease progression (168). Research has found that NF-κB activity is significantly elevated in the joint tissues of osteoarthritis patients and positively correlates with disease severity. Inhibiting the NF-κB signaling pathway can effectively reduce OA inflammation and joint damage, indicating its important regulatory role in the pathogenesis of OA mediated by necroptosis (169, 170).

**Figure 4:**
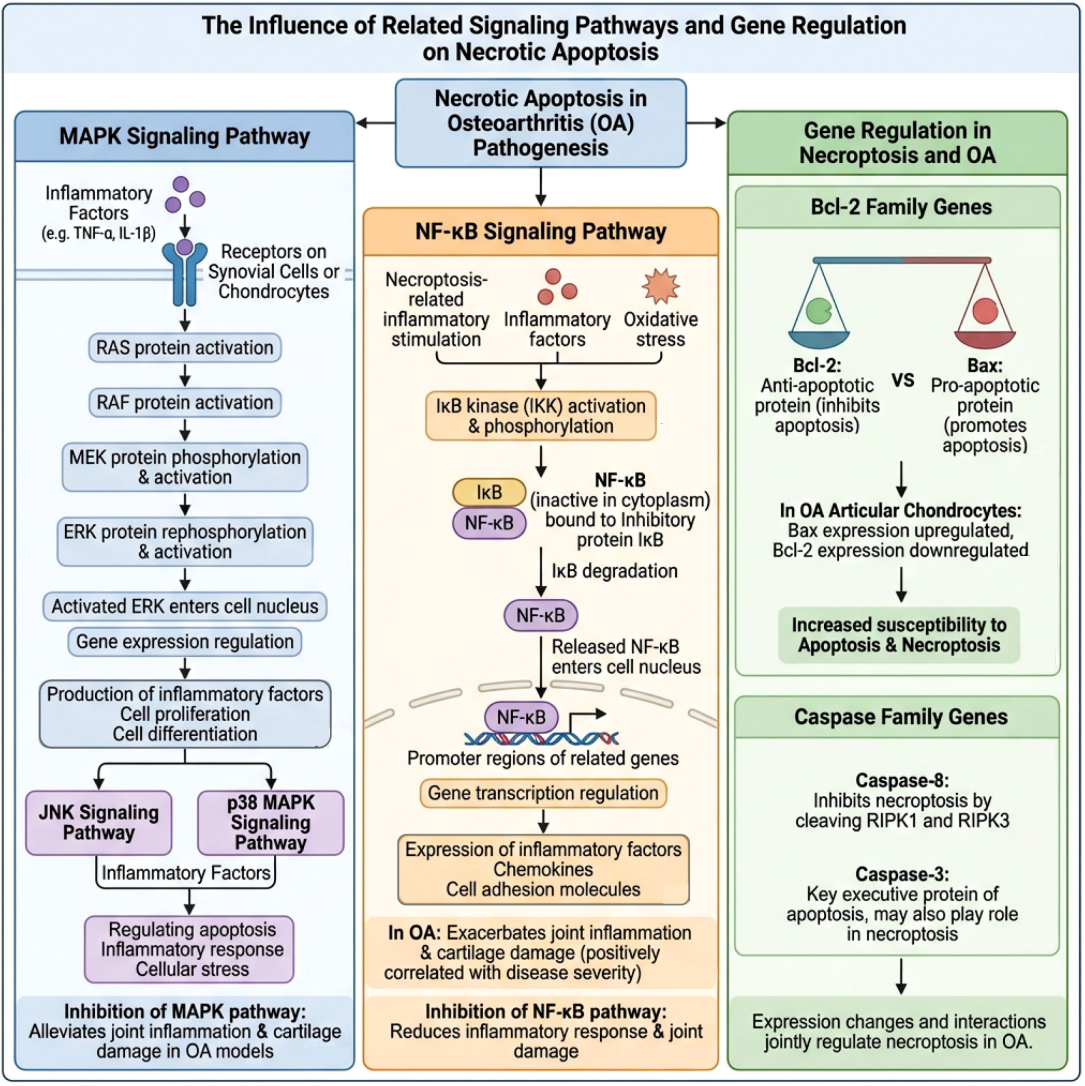
The schematic diagram illustrates the effects of related signaling pathways and gene regulation on necroptosis in osteoarthritis. On the left is the MAPK signaling pathway. After inflammatory factors such as TNF-α and IL-1β bind to surface receptors on synovial cells or chondrocytes, they sequentially activate the RAS–RAF–MEK–ERK signaling cascade. This cascade transmits signals to the nucleus to regulate gene expression, thereby promoting the production of inflammatory factors as well as cell proliferation and differentiation. Additionally, the JNK and p38 MAPK pathways participate in regulating apoptosis, inflammatory responses, and cellular stress. Overall, inhibiting the MAPK pathway can alleviate joint inflammation and cartilage damage in osteoarthritis. In the middle is the NF-κB signaling pathway. Necroptosis-related stimuli, inflammatory factors, and oxidative stress activate the IKK complex, which causes phosphorylation and degradation of IκB; this releases NF-κB into the nucleus, where it binds to target gene promoters and induces the expression of various inflammatory factors, chemokines, and adhesion molecules. These events exacerbate synovitis and cartilage destruction in osteoarthritis. Inhibiting NF-κB can reduce inflammatory responses and joint damage. On the right is gene regulation related to necroptosis and osteoarthritis. Within the Bcl-2 family, an imbalance between the anti-apoptotic protein Bcl-2 and the pro-apoptotic protein Bax—characterized by upregulation of Bax and downregulation of Bcl-2 in osteoarthritic chondrocytes—increases the susceptibility of cells to apoptosis and necroptosis. Among the Caspase family, Caspase-8 inhibits necroptosis by cleaving RIPK1 and RIPK3, while Caspase-3 is a key executioner protein in apoptosis and may also participate in necroptosis. The expression and interaction of these genes collectively determine the extent of necroptosis in osteoarthritis.

In addition to the aforementioned signaling pathways, several key genes are involved in the regulation of necroptosis and OA. For example, the B-cell lymphoma-2 (Bcl-2) family of genes play a significant role in the regulation of apoptosis (171). Bcl-2 is an anti-apoptotic protein that inhibits apoptosis, while Bax is a pro-apoptotic protein that promotes apoptosis (172). In OA, an imbalance in the expression of Bcl-2/Bax may make cells more susceptible to entering irreversible death pathways. Early-stage changes may manifest as fluctuations under stress conditions, while mid-to-late stages often exhibit a sustained imbalance characterized by “upregulation of Bax and downregulation of Bcl-2,” increasing cell death susceptibility and providing a basis for the coupling of necroptosis and inflammatory damage (173, 174). Furthermore, some apoptosis-related genes, such as members of the caspase family, are also involved in the regulation of necroptosis. Caspase-8 can inhibit necroptosis by cleaving RIPK1 and RIPK3; thus, its inhibitory effect in the early stage of OA helps limit the initiation of necroptosis. However, in the mid-to-late stages, when inflammation persists, energy metabolism is impaired, or stress accumulates, and this inhibitory effect weakens, RIPK1/RIPK3-mediated necroptosis is more easily disinhibited and persistently activated. Caspase-3, as a key executioner protein in apoptosis, may also play a role in the switch between necroptotic and apoptotic pathways (175, 176). Overall, the expression changes and interactions of these signaling pathways and key genes collectively determine the initiation intensity and persistence of necroptosis at different stages of OA, thereby influencing disease progression (**Figure 4**).

## 6 Therapeutic strategies targeting necroptosis

Necroptosis plays a key role in the pathogenesis of osteoarthritis (OA). By inhibiting the necroptosis signaling pathway, the death of chondrocytes and synoviocytes can be reduced. Inflammatory responses can be alleviated, thereby delaying the progression of osteoarthritis. Inhibiting the activity of key necroptosis-related proteins is an important therapeutic strategy. RIPK1, RIPK3, and MLKL are core molecules in the necroptosis signaling pathway, and inhibiting their activity can effectively block the occurrence of necroptosis. It is important to recognize that necroptosis also participates in the body’s anti-infection defense and the maintenance of tissue homeostasis. Systemic and long-term intervention targeting RIPK1/RIPK3/MLKL may pose potential risks such as infection risks, pathway crosstalk, and impaired tissue repair. Therefore, while focusing on its potential benefits, it is also necessary to address associated safety concerns and challenges in clinical translation.

### 6.1 RIP1 inhibitors

RIP1 plays a critical initiating role in the necroptosis signaling pathway, and its activation is a key step in the occurrence of necroptosis. Therefore, RIP1 inhibitors have become an important research direction for inhibiting necroptosis (177). Necrostatin-1 is the earliest discovered and relatively well-studied RIP1 inhibitor (178). Nec-1 is a type III kinase inhibitor that effectively blocks RIP1-RIP3-MLKL signal transduction by inhibiting RIP1 phosphorylation, thereby suppressing cellular necroptosis (179). In cell experiments, following Necrostatin-1 treatment, indicators such as cell viability, lactate dehydrogenase (LDH) release, and the expression of necroptosis-related proteins were measured. These results demonstrated that cell viability significantly increased, LDH release decreased, and the phosphorylation levels of necroptosis execution proteins like RIPK3 and MLKL were reduced (180). In animal experiments, a knee osteoarthritis mouse model was established, and Necrostatin-1 was administered via intraperitoneal injection. Joint imaging examinations (such as Micro-CT and MRI) and histopathological analyses (HE staining and immunohistochemistry) revealed that joint cartilage damage in mice was alleviated, the degree of joint inflammation decreased, and synovial hyperplasia and inflammatory cell infiltration were reduced (181). This indicates that Necrostatin-1 can effectively inhibit necroptosis both *in vitro* and *in vivo*, showing certain therapeutic effects on osteoarthritis. In addition to Necrostatin-1, some novel RIP1 inhibitors are under development, such as GSK’963, GSK’872, PK68, RIPA-56, 6E11, SAR443060, and GSK2982772, which have been proven effective for various diseases (182). For example, the compound GSK’872 has shown highly selective inhibitory effects on RIP1 kinase activity in preclinical studies (183). The emergence of these novel RIP1 inhibitors provides more options and hope for targeted therapy of OA, potentially further improving efficacy and reducing adverse reactions. However, it should be noted that RIP1 is also involved in inflammation regulation and cell survival maintenance, and most inhibitors are still in the preclinical or early trial stages. Long-term systemic application may pose immune-related risks and potential off-target effects, and appropriate routes of administration and dosing regimens need further clarification.

### 6.2 RIP3 inhibitors

RIP3 plays a pivotal role in the necroptosis signaling pathway, bridging upstream and downstream events. Upon activation, RIP3 phosphorylates MLKL, thereby triggering cellular necroptosis. Consequently, RIP3 inhibitors have emerged as crucial agents for inhibiting necroptosis. Li et al. found that the B-Raf inhibitor (VE600), traditionally used to treat metastatic melanoma, can also inhibit RIP3 (184). For example, compounds such as dabrafenib competitively inhibit the binding of ATP to the RIP3 enzyme, reduce RIP3-mediated MLKL phosphorylation, and disrupt the interaction between RIP3 and MLKL (185). Some anticancer drugs, such as ponatinib, can simultaneously inhibit RIP1 and RIP3, thereby suppressing necroptosis (186). Additionally, drugs like HS-1371, AZD5423, and TAK-632 have been shown to effectively inhibit RIP3 (187, 188). These findings suggest that by inhibiting the expression or kinase activity of RIP3, the processes of necroptosis, joint injury, and inflammatory response in animal models of osteoarthritis can be effectively alleviated, indicating potential therapeutic value for OA. However, it should be noted that many current RIP3 inhibitors are derived from antitumor or multi-target kinase inhibitors. Their systemic adverse reaction profiles do not fully align with the safety requirements for chronic OA treatment. How to reduce toxicity and optimize dosing strategies while ensuring target selectivity remains a critical issue to be addressed for their clinical translation.

### 6.3 MLKL inhibitors

As the ultimate executor of necroptosis, mixed lineage kinase domain-like protein (MLKL) is instrumental in the necroptotic pathway; consequently, the development of MLKL inhibitors has emerged as a significant avenue for the suppression of necroptosis. Necrosulfonamide (NSA) is one of the extensively investigated MLKL inhibitors (189). In a mouse model of knee osteoarthritis, intraperitoneal injection of NSA was found to reduce articular cartilage damage, ameliorate joint space narrowing, and decrease osteophyte formation. It was also observed that inflammatory cell infiltration in synovial tissue decreased, the number of chondrocytes undergoing necroptosis reduced, and the degradation of the extracellular matrix was alleviated (181). By screening the kinase inhibitor library released by GSK, researchers identified GW806742X (also known as SYN-1215) as an MLKL inhibitor (190). GW806742X exhibits multi-pharmacological effects on necroptosis because it non-specifically binds to kinases (such as RIPK1) rather than specifically targeting MLKL (191). Given that MLKL is at the terminal execution stage of necroptosis, the long-term impact of inhibiting its activity on the body’s anti-infection capacity, tissue repair, and outcomes of other inflammatory diseases is not yet fully understood. Furthermore, some candidate drugs have limitations such as species differences and insufficient target specificity. Therefore, their application in OA still requires more systematic preclinical and early clinical studies for validation (**Table 1**).

**Table 1:** Mechanism of Action of necrotic apoptosis inhibitors targeting necroptosis in the treatment of OA.

Target spot	Representative inhibitors	Mechanism	Therapeutic effect	References
RIPK1				
	Necrostatin-1	Type III kinase inhibitors block the phosphorylation of RIP1 and the RIP1-RIP3-MLKL signaling pathway	Enhance cell viability, reduce LDH release, and lower the phosphorylation level of RIPK3/MLKL	(180, 181)
	GSK’872	Type III kinase inhibitors block the phosphorylation of RIP1 and the RIP1-RIP3-MLKL signaling pathway	Alleviate cartilage injury, inflammation, synovial hyperplasia and inflammatory cell infiltration in OA mice	(183)
RIPK3				
	Dabrafenib	Competitively inhibit ATP binding, block the activation of RIP3 and its phosphorylation effect on MLKL	Reduce MLKL phosphorylation mediated by RIP3, inhibit necrotic apoptosis, and alleviate joint injury and inflammatory response in OA	(185)
	Ponatinib	Competitively inhibit ATP binding, block the activation of RIP3 and its phosphorylation effect on MLKL	Reduce MLKL phosphorylation mediated by RIP3, inhibit necrotic apoptosis, and alleviate joint injury and inflammatory response in OA	(186)
MLK				
	NSA	The final execution step of inhibiting MLKL activation and preventing necrotic apoptosis	Reduce articular cartilage damage and decrease the infiltration of synovial inflammatory cells	(181)
	GW806742X	Non-specifically binds to kinases (such as RIPK1)	Reduce articular cartilage damage and decrease the infiltration of synovial inflammatory cells	(191)

From a clinical perspective, the current standard treatment for OA primarily includes oral or topical non-steroidal anti-inflammatory drugs, intra-articular injections of corticosteroids or hyaluronic acid, structure- or symptom-modifying drugs, and end-stage joint replacement surgery. These strategies focus mainly on alleviating pain and improving joint function, with limited direct intervention in structural cartilage damage and disease progression. In contrast, RIPK1/RIPK3/MLKL inhibitors targeting necroptosis aim to protect cartilage and synovium from the source of cell death and inflammatory amplification, theoretically aligning more closely with “disease-modifying” interventions. They are expected to serve as a mechanistic complement to existing symptomatic treatments. However, related drugs are still in the preclinical or early exploratory stages and are unlikely to replace current standard treatments in the short term. They hold greater promise for providing more precise treatment options in the future, particularly for OA subtypes with high inflammatory burden or elevated necroptosis markers, when combined with traditional drugs or rehabilitation programs.

## 7 Current research limitations and future research directions

While a fundamental comprehension of necroptosis within the context of OA has been achieved, significant challenges and unresolved issues persist in this domain. Regarding the exploration of pathogenesis, although a connection between necroptosis and OA has been established, along with the identification of several principal pathways, the intricate molecular regulatory networks governing necroptosis in OA remain inadequately understood. For instance, despite the recognition of the pivotal role of the RIPK1-RIPK3-MLKL pathway in necroptosis, the interactions and synergistic mechanisms that exist between this pathway and other signaling pathways necessitate further investigation. Moreover, in cellular studies, the activation and modulation of necroptosis-related signaling pathways exhibit variability depending on the specific cell types and experimental conditions utilized, complicating the comprehensive understanding of its molecular underpinnings. Additionally, research exploring the interplay between necroptosis and other forms of cell death, such as apoptosis and autophagy, in the context of OA is notably limited, and elucidating the ways in which these processes influence and co-regulate the progression of OA remains an urgent challenge that demands resolution.

In the realm of therapeutic research, the majority of contemporary treatment strategies aimed at targeting necroptosis remain confined to laboratory investigations and preclinical animal studies. These approaches encounter significant obstacles when attempting to progress from fundamental research to clinical practice. With respect to drug development, although several small-molecule inhibitors that focus on critical pathways associated with necroptosis have been synthesized, their pharmacokinetic profiles, safety, and effectiveness *in vivo* require additional verification. It is noteworthy that pharmaceuticals demonstrating efficacy in animal models may present divergent responses during human clinical trials, potentially leading to complications such as drug toxicity and immunogenicity. Furthermore, while gene therapy holds considerable promise, it is not without its own set of challenges, particularly regarding the safety of gene delivery systems. Commonly utilized viral vectors may incite immune reactions and carry the risk of insertional mutagenesis, which could ultimately result in carcinogenesis. The efficiency of gene transfection also poses a significant hurdle; achieving reliable delivery of therapeutic genes into target cells while ensuring stable expression remains a formidable technical challenge in the field of gene therapy. Moreover, clinical studies frequently suffer from limitations such as small sample sizes, brief study durations, and insufficient long-term follow-up, which restricts our comprehension of the enduring efficacy and safety associated with treatment modalities founded on necroptosis.

In light of the challenges and issues present in current research, there are several important research directions for necroptosis in the field of OA in the future. In terms of pathogenesis research, further exploration of the molecular mechanisms of necroptosis is needed. Utilizing multi-omics technologies, such as transcriptomics, proteomics, and metabolomics, to comprehensively analyze the changes in genes, proteins, and metabolites related to necroptosis in OA will help construct a more complete molecular regulatory network. Strengthening the study of the interaction mechanisms between necroptosis and other forms of cell death will clarify their synergistic or antagonistic relationships in the pathogenesis of OA, providing a more comprehensive theoretical basis for understanding the pathological processes of OA.

**Figure 5:**
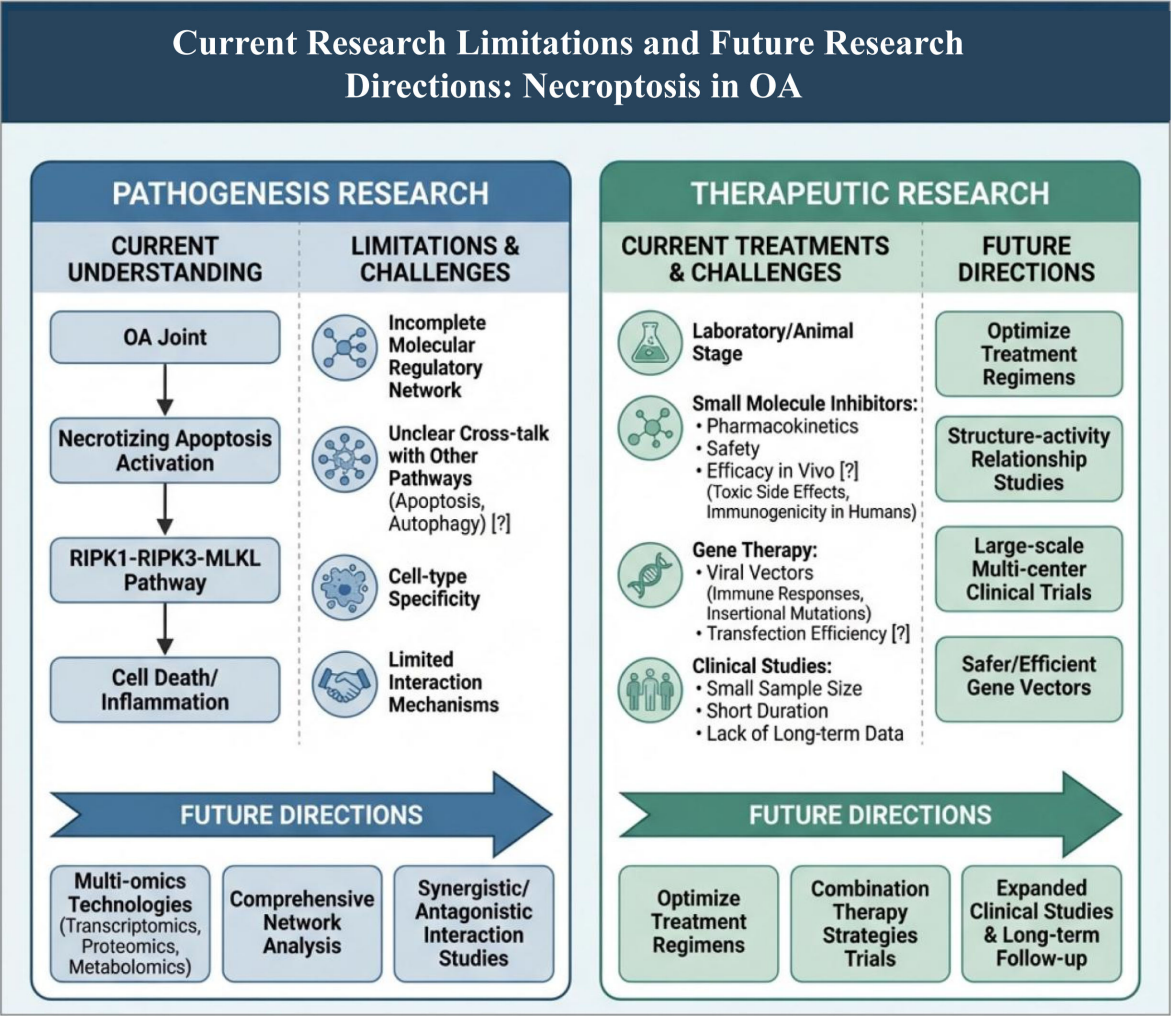
This article illustrates the limitations and future directions of necroptosis research in osteoarthritis. Regarding the study of pathogenesis, the current understanding progresses from the OA joint to the activation of necroptosis, involving the RIPK1–RIPK3–MLKL pathway, which leads to cell death and inflammation. However, challenges remain, including incomplete molecular regulatory networks, unclear crosstalk with apoptosis, autophagy, and other pathways, limited understanding of cell type specificity, and poorly defined mechanisms of pathway interactions. Future research should integrate multi-omics technologies—such as transcriptomics, proteomics, and metabolomics—to construct a more comprehensive regulatory network. Additionally, systematic studies are needed to explore the synergistic and antagonistic relationships among different modes of cell death, including necroptosis, apoptosis, and autophagy. In terms of therapeutic research, existing treatments face challenges related to pharmacokinetics and safety, *in vivo* efficacy, vector immunogenicity and insertion mutation risks, as well as small clinical sample sizes and short follow-up durations. These challenges are observed across stages ranging from animal experiments and small-molecule inhibitors to gene therapy and clinical trials. Moving forward, it is necessary to optimize treatment and dosing regimens and to conduct structure-activity relationship studies to improve drug design. Furthermore, designing safer and more effective gene vectors is crucial. The efficacy of interventions targeting necroptosis should be validated through combination therapy strategies and large-scale, multi-center, long-term follow-up clinical trials.

In therapeutic research, further optimization of treatment plans is necessary. In drug development, in-depth studies on the structure-activity relationships of small-molecule inhibitors should be conducted, and through structural modifications and optimizations, the efficacy and safety of drugs can be improved. Large-scale, multi-center clinical trials should be conducted to validate the efficacy and safety of drugs, accelerating the clinical translation process. For gene therapy, the development of safer and more efficient gene vectors is needed to improve gene transfection efficiency and targeting. Exploring combination therapy strategies that integrate treatment methods targeting necroptosis with traditional OA treatments (such as pharmacological therapy, physical therapy, surgical therapy, etc.) can enhance synergistic effects and improve therapeutic outcomes. Additionally, strengthening clinical research, expanding sample sizes, extending study durations, and conducting long-term follow-up observations will comprehensively assess the efficacy and safety of treatment methods based on necroptosis in OA patients, providing more reliable evidence for clinical treatment (**Figure 5**).

## 8 Conclusion

Necroptosis, a novel form of programmed cell death, plays a key role in the pathogenesis of OA. To facilitate a concise overview of this article, we propose a simplified “inflammation–necroptosis–tissue damage” summary model: In the early and progressive stages of OA, inflammatory factors such as TNF-α and IL-1β, along with abnormal mechanical stress, jointly induce a local stress response and activate the necroptosis pathway, including RIPK1/RIPK3/MLKL. Subsequently, chondrocytes and synovial cells undergo necroptosis, resulting in cell membrane rupture and leakage of cellular contents that release DAMPs and matrix fragments. This process is accompanied by enhanced catabolic activities, such as MMPs/ADAMTS, promoting extracellular matrix degradation and cartilage structural damage. These DAMPs and degradation products further activate inflammatory signals and promote immune cell recruitment, amplifying inflammatory networks such as TNF-α/IL-1β in a feedback loop, thereby forming a self-reinforcing cycle of “inflammatory stimulation → necroptosis → release of DAMPs/matrix fragments → amplification of inflammation and aggravation of tissue damage,” ultimately accelerating cartilage degeneration, persistent synovial inflammation, and joint structural destruction. Based on this model, necroptosis is not only the “effector endpoint” in an inflammatory environment but also serves as a critical hub connecting inflammatory load and structural progression through DAMP-mediated feedback amplification. Therefore, targeting necroptosis-related signaling pathways or key molecules (such as RIPK1, RIPK3, MLKL) theoretically has the potential to interrupt the aforementioned vicious cycle, reduce cell death and inflammatory amplification from the source, and provide OA with strategies that have greater disease-modifying potential beyond the existing treatment system primarily focused on analgesia and functional improvement. Overall, systematically elucidating the role of necroptosis in the pathogenesis of OA and its coupling relationship with the inflammation–tissue damage closed loop holds significant theoretical and clinical application value for optimizing stratified interventions and advancing targeted therapy translation.
